# Overexpression of the kiwifruit *SVP3* gene affects reproductive development and suppresses anthocyanin biosynthesis in petals, but has no effect on vegetative growth, dormancy, or flowering time

**DOI:** 10.1093/jxb/eru264

**Published:** 2014-06-19

**Authors:** Rongmei Wu, Tianchi Wang, Tony McGie, Charlotte Voogd, Andrew C. Allan, Roger P. Hellens, Erika Varkonyi-Gasic

**Affiliations:** ^1^The New Zealand Institute for Plant & Food Research Limited (Plant & Food Research) Mt Albert, Private Bag 92169, Auckland 1142, New Zealand; ^2^The New Zealand Institute for Plant & Food Research Limited (Plant & Food Research) Palmerston North, Private Bag 11600, Palmerston North 4442, New Zealand; ^3^School of Biological Sciences, University of Auckland, Private Bag 92019, Auckland, New Zealand

**Keywords:** *Actinidia*, bud break, dormancy, flowering, kiwifruit, MYB, Nicotiana, petal colour, SVP.

## Abstract

SVP-like MADS domain transcription factors have been shown to regulate flowering time and both inflorescence and flower development in annual plants, while having effects on growth cessation and terminal bud formation in perennial species. Previously, four *SVP* genes were described in woody perennial vine kiwifruit (*Actinidia* spp.), with possible distinct roles in bud dormancy and flowering. Kiwifruit *SVP3* transcript was confined to vegetative tissues and acted as a repressor of flowering as it was able to rescue the *Arabidopsis svp41* mutant. To characterize kiwifruit *SVP3* further, ectopic expression in kiwifruit species was performed. Ectopic expression of *SVP3* in *A. deliciosa* did not affect general plant growth or the duration of endodormancy. Ectopic expression of *SVP3* in *A. eriantha* also resulted in plants with normal vegetative growth, bud break, and flowering time. However, significantly prolonged and abnormal flower, fruit, and seed development were observed, arising from SVP3 interactions with kiwifruit floral homeotic MADS-domain proteins. Petal pigmentation was reduced as a result of *SVP3*-mediated interference with transcription of the kiwifruit flower tissue-specific R2R3 MYB regulator, *MYB110a*, and the gene encoding the key anthocyanin biosynthetic step, *F3GT1*. Constitutive expression of *SVP3* had a similar impact on reproductive development in transgenic tobacco. The flowering time was not affected in day-neutral and photoperiod-responsive *Nicotiana tabacum* cultivars, but anthesis and seed germination were significantly delayed. The accumulation of anthocyanin in petals was reduced and the same underlying mechanism of *R2R3 MYB NtAN2* transcript reduction was demonstrated.

## Summary

Overexpression of *SVP3* affects kiwifruit flower and fruit development. The reduced petal pigmentation results from interference with transcription of the kiwifruit flower tissue-specific R2R3 MYB regulator.

## Introduction

Plant MADS-domain transcription factors act as key regulators of many developmental processes (Smaczniak *et al.*, 2012). The STMADS subfamily of MADS-box genes are predominantly expressed in vegetative tissues and play roles in vegetative development and transition to flowering in diverse plant species (Carmona *et al.*, 1998; Hartmann *et al.*, 2000; Mao *et al.*, 2000; Yu *et al.*, 2002; Masiero *et al.*, 2004; Aswath and Kim, 2005; Liu *et al.*, 2007; Trevaskis *et al.*, 2007; He *et al.*, 2010; Cohen *et al.*, 2012; Cooke *et al.*, 2012).

Relatively small changes in sequence can have a major effect on STMADS gene function. In *Arabidopsis thaliana*, the two STMADS genes *SVP* and *AGL24* act in an opposite manner despite their close homology. SVP plays a key role as a repressor of the transition to flowering; a single amino acid substitution causes the loss of function resulting in an early flowering phenotype (Hartmann *et al.*, 2000; Méndez-Vigo *et al.*, 2013). In contrast, AGL24 acts as a promoter of flowering because its inactivation results in late flowering (Yu *et al.*, 2002). Both proteins have further roles during early stages of flower development to repress expression of class B and C floral homeotic genes and maintain floral meristem identity (Gregis *et al.*, 2009). Protein interactions and formation of complexes with other MADS-domain proteins are essential for STMADS function (Liu *et al.*, 2009; Gregis *et al.*, 2013). Interaction with APETALA1 (AP1) in particular resulted in floral reversion phenotypes observed in transgenic *Arabidopsis* upon ectopic expression of *SVP*-like genes (Liu *et al.*, 2007; Gregis *et al.*, 2008; Fornara *et al.*, 2008; Wu *et al.*, 2012; Jaudal *et al.*, 2013). For these and perhaps other reasons, the function of STMADS observed upon ectopic expression in *Arabidopsis* might be different from their function in the species of origin (Trevaskis *et al.*, 2007; Jaudal *et al.*, 2013).

In woody perennial plants, the STMADS subfamily genes have been reported to act as growth inhibitors, which correlated with maintenance of plant dormancy (Mazzitelli *et al.*, 2007; Bielenberg *et al.*, 2008; Diaz-Riquelme *et al.*, 2009; Horvath *et al.*, 2010; Li *et al.*, 2010; Sasaki *et al.*, 2011; Yamane *et al.*, 2011). Deletion of the six *SVP*-like *DORMANCY-ASSOCIATED MADS-BOX* (*DAM*) genes in peach (*Prunus persica*) resulted in failure to enter dormancy under cold or short-day induction, and the expression of a subset of these genes was elevated during endodormancy (Bielenberg *et al.*, 2008; Li *et al.*, 2009; Yamane *et al.*, 2011). Similarly, a negative correlation of expression with endodormancy release was observed for six tandemly arrayed *DAM* genes predicted to act as transcriptional repressors in Japanese apricot (*Prunus mume*) (Yamane *et al.*, 2008; Sasaki *et al.*, 2011). Ectopic expression of one of these genes in transgenic poplar resulted in premature growth cessation and terminal bud set, demonstrating the role in growth inhibition (Sasaki *et al.*, 2011). However, neither of these genes was functionally characterized by ectopic expression in the plant species of origin.

In the kiwifruit species *Actinidia chinensis* and *A. deliciosa*, four *SVP* genes (*SVP1–SVP4*) have been identified, with potentially distinct roles in bud dormancy and flowering (Wu *et al.*, 2012). Their expression was mostly confined to vegetative tissues, with *SVP3* showing the highest relative expression. *SVP1*, *SVP2*, and *SVP4* were elevated in buds over the winter dormancy period. In contrast, *SVP3* accumulation in buds did not demonstrate seasonal changes, but delayed flowering in transgenic *Arabidopsis* and was able to rescue the *Arabidopsis svp41* mutant. Ectopic expression resulted in floral reversion phenotypes, caused by the strong capacity for heterodimerization with *Arabidopsis* MADS box proteins responsible for flower development (Wu *et al.*, 2012).

In the current study, functional analysis of *SVP3* was performed using ectopic transgenic analysis in kiwifruit species *A. deliciosa* and *A. eriantha*, which have different chilling requirements and bloom time, and a model plant species tobacco (*Nicotiana tabacum*). Normal vegetative growth and floral transition, but abnormal flower development, reduced petal pigmentation, and abnormal fruit and seed development were observed. The underlying mechanism of reduced anthocyanin accumulation in petals was examined. Based on the results obtained, the biological function of *SVP3* is discussed.

## Materials and methods

### Gene isolation and vector construction

The *A. chinensis SVP3* coding sequence was cloned under the control of the *Cauliflower mosaic virus* (CaMV) 35S promoter (*35S:SVP3*) into the pART277 binary vector (Gleave, 1992) as previously described (Wu *et al.*, 2012). The resulting plasmid was transformed into *Agrobacterium tumefaciens* strain EHA105 for transformation into *A. deliciosa* and *A. eriantha*, and into GV3101 for transformation into *N. tabacum*. A construct with a reporter gene *uidA* (*GUS*; β-gluronidase) under the control of the CaMV 35S promoter (*35S:GUS*) in appropriate *Agrobacterium* strains was used to transform control plants (Wang *et al.*, 2006). *Actinidia eriantha SVP3* (GenBank accession no. KJ123703) was amplified from the bud cDNA using gene-specific oligonucleotide primers, 5’-ATGGCGAGAGAGAAGATCAAGA-3’ (forward) and 5’-TGGTGTGACATTTCAAGTTCG-3’ (reverse). The predicted amino acid sequence alignment with *A. deliciosa* and *A. chinensis* SVP3 homologues was performed using Vector NTI version 9.0. (Invitrogen) Clustal W (opening 15, extension penalty 0.3).

### Plant transformation and growth conditions

The transformation procedures for *A. deliciosa* and *A. eriantha* were as previously described (Wang *et al.*, 2006, 2007). Once roots were established, transgenic plants were transferred to soil and grown in the containment glasshouse at the Plant & Food Research, Mt Albert, Auckland, New Zealand for 18 months. Flowering time and floral phenotype were assessed in the following spring season. *Nicotiana tabacum* ‘Maryland Mammoth’ and ‘Samsun’ transformations were carried out on young leaf discs excised from *in vitro* grown shoots (Horsch *et al.*, 1985). Transgenic tobacco plants were grown in a glasshouse at 20 °C under long-day (16/8h light/dark; for ‘Samsun’) or short-day conditions (8/16h light/dark; for ‘Maryland Mammoth’). The seeds from these transgenic plants were collected and germinated on half-strength Murashige and Skoog (MS) agar medium (Murashige and Skoog, 1962) supplemented with 50 μg ml^–1^ kanamycin. Following the segregation tests, two homozygous lines of both cultivars at T_2_ generations were used for flowering time and floral phenotype assessment.

### RNA extraction and expression studies

*Actinidia eriantha* root, stem, leaf, axillary bud, shoot apex, flower, and fruit tissue collection was carried out on vines growing in the glasshouse at the Plant & Food Research, Mt Albert, Auckland, New Zealand, during the spring and summer season of 2011–2012. *Actinidia chinensis* root, stem, leaf, flower, fruit, and axillary and apical bud collection was carried out on vines growing at the Plant & Food Research orchard near Kerikeri, New Zealand, during the spring and summer season of 2005–2006.

Total RNA was extracted from kiwifruit tissue as previously described (Chang *et al.*, 1993). Total RNA was isolated from tobacco flowers using the Trizol reagent (Invitrogen). A 5 μg aliquot of total RNA was treated with DNase I (Ambion) and reverse-transcribed at 37 °C using the BluePrint^®^ Reagent kit for reverse transcription–PCR (RT–PCR; TaKaRa) according to the manufacturer’s instructions. Amplification and quantification were carried out using the LightCycler^®^ 480 System and LightCycler^®^ 480 SYBR Green I Master Mix (Roche Diagnostics). A minimum of three technical replicate reactions were performed and a non-template control was included in each run. Thermal cycling conditions were 95 °C for 5min, followed by 50 cycles of 95 °C for 10 s, 60 °C for 10 s, and 72 °C for 20 s, and a melting temperature cycle, with constant fluorescence data acquisition from 65 °C to 95 °C. The data were analysed using the Target/Reference ratio calculated with the LightCycler^®^ 480 software 1.5 (Roche Diagnostics). The expression was normalized to previously characterized reference genes, kiwifruit *actin* (Wu *et al.*, 2012) and tobacco *Ntα-Tub1* (Pattanaik *et al.*, 2010).

Oligonucleotide primers for kiwifruit *SVP3*, *actin*, anthocyanin pathway genes, and regulatory genes *MYB110* and *MYB10* were as previously described (Montefiori *et al.*, 2011; Wu *et al.*, 2012; Fraser *et al.*, 2013). Oligonucleotide primers for tobacco *NtAn1a*, *NtAN1b*, *NtAn2*, and *Ntα-Tub1* were as described by Bai *et al.* (2011) and Pattanaik *et al.* (2010).

### Biochemical analyses

For chlorophyll quantification, petals collected at visually similar developmental stages were frozen and ground in liquid nitrogen. Ground tissues were suspended in 80% acetone with 2.5M phosphate buffer pH 7.5 and incubated for 1h in the dark, followed by centrifugation at 16 000 *g* for 10min. The absorbance of 200 μl of the supernatant was measured spectrophotometrically at 663nm and 646nm (SpectraMax 384, Molecular Devices, Sunnyvale, CA, USA). The total chlorophyll (C_a+b_) concentration was calculated using the equation C_a+b_ (μg ml^–1^) =7.15*A*_663_+18.71*A*_646_ (Lichtenthaler and Rinderle, 1988).

Anthocyanins and flavonoids were extracted from lyophilized samples and analysed by high-performance liquid chromatography (HPLC) and liquid chromatography–mass spectrometry (LC-MS) as described previously (McGhie *et al.*, 2011; Dare *et al.*, 2013). Quantification of cyanidin 3-*O*-galactoside was achieved by calibration at 520nm by HPLC analysis. The peaks were identified by a comparison of retention times with authentic standards of cyanidin 3-*O*-galactoside (Polyphenols Laboratories, Sandnes, Norway).

### Microscopic analyses

Tobacco petal epidermis was obtained by careful peeling and observed under a light microscope (Olympus Vanox AHT3, Olympus Optical Co. Ltd, Tokyo, Japan). Photographs were taken using a CoolSNAP Colour camera system (Roper Scientific Ltd, Tucson, AZ, USA).

### Yeast two-hybrid assay

The full-length open reading frame of kiwifruit *SVP* genes and kiwifruit *AGAMOUS* (*AG*), *FRUITFULL* class (*FUL* and *FUL*-like), *SEPALLATA* class (*SEP1*, *SEP3*, and *SEP4*), *APETALA3* (*AP3*), *PISTILLATA* (*PI*), and *CENTRORADIALIS* (*CEN*) were amplified and cloned as described previously (Varkonyi-Gasic *et al.*, 2011, 2013; Wu *et al.*, 2012). The bait vector and prey vector were mated and selected on synthetic complete (SC) plates lacking leucine and tryptophan. The affinity of two protein interactions was determined on selective SC medium lacking leucine, tryptophan, and histidine, and in the presence of 1, 3, and 5mM 3-aminotriazole (3AT) at 30 °C. All assayed proteins were tested for autoactivation, homodimerization, and heterodimerization capacity. The strength of interactions was compared with control plasmids of pEXP™32/Krev1 with pEXP™22/RalGDS-wt, pEXP™22/RalGDS-m1, and pEXP™22/RalGDS-m2 (Invitrogen).

## Results

### Overexpression of *SVP3* has no significant effect on growth or dormancy in *A. deliciosa*

**Fig. 1.**
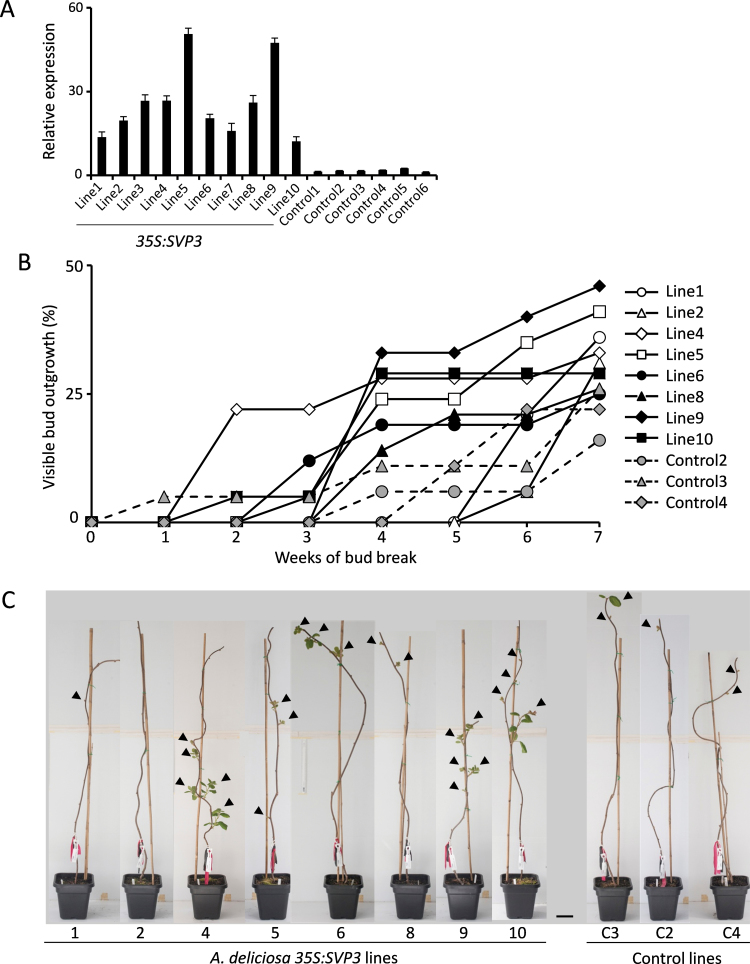
Overexpression of *SVP3* in *Actinidia deliciosa*. (A) Relative expression of *SVP3* in *35S:SVP3* and control plants. The expression was normalized against kiwifruit *actin*. Error bars represent the standard error (SE) of four replicate reactions. (B) Bud break time measured as a percentage of total buds with visible shoot outgrowth. (C) A subset of *35S:SVP3* and control *A. deliciosa* plants, photographed on the same day in spring. Arrowheads indicate positions of visible bud break and shoot outgrowth. Scale bar=10cm.

To investigate the role of kiwifruit *SVP3*, transgenic *A. deliciosa* was generated using the *SVP3* cDNA driven by the CaMV 35S promoter (Wu *et al.*, 2012). Ten independent transgenic lines with high *SVP3* transgene expression levels were generated and compared with six control lines (Fig. 1A). Monitoring during callus formation, plantlet growth in tissue culture, and upon transfer to soil revealed no obvious difference between *35S:SVP3* lines and control lines. Eight *35S:SVP3* and three control lines were further monitored for spring bud break. The number of breaking buds or developing shoots was recorded weekly and is presented as a percentage of total buds. Large variation in the time of the first visible bud break, positions of breaking buds, number of developing branches, and rate of shoot outgrowth was observed for all lines; however, the first visible bud break time of the *35S:SVP3* lines was in the range recorded for control lines and was followed by normal growth of multiple branches (Fig. 1B, C). Therefore, it was concluded that *SVP3* does not have a significant effect on growth and dormancy in *A. deliciosa* under glasshouse conditions.

### *SVP3* affects reproductive development in transgenic A. eriantha

**Fig. 2.**
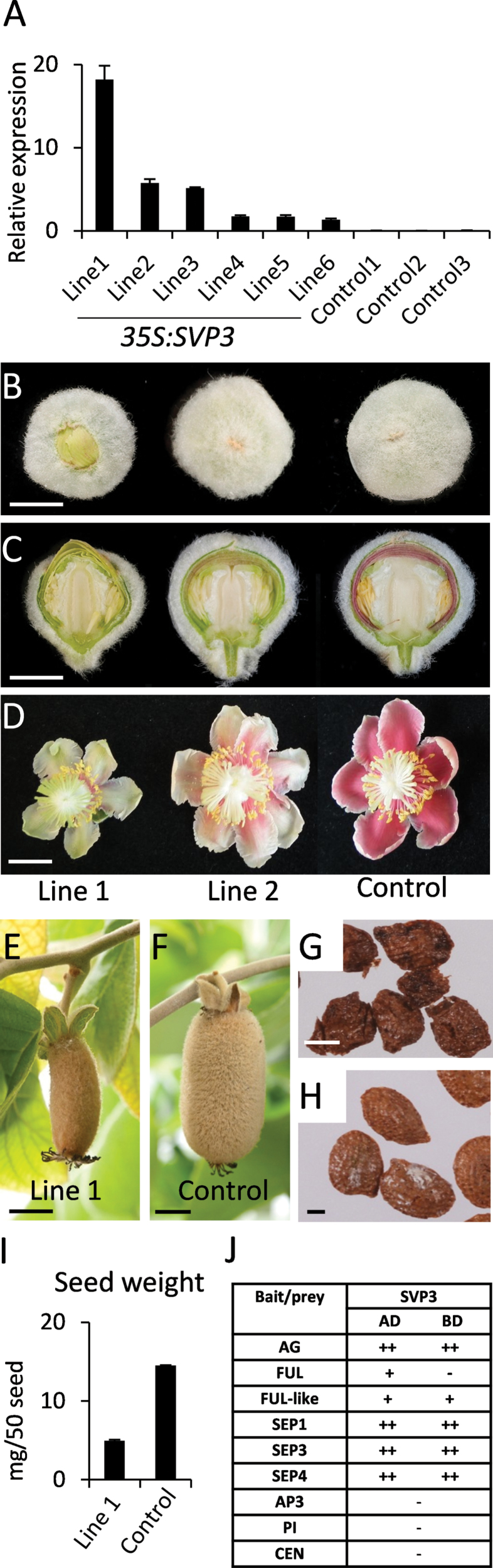
Overexpression of *SVP3* in *Actinidia eriantha*. (A) Relative expression of *SVP3* in *35S:SVP3* and control plants. The expression was normalized against kiwifruit *actin*. Error bars represent the standard error (SE) of four replicate reactions. (B–D) Morphology of flower buds (B, C) and fully open flowers (D). (E, F) Fruit morphology. (G–I) Seed morphology (G, H) and weight (I). Error bars represent the standard error (SE), *n*=3. Scale bars=5mm (B, C), 10mm (D–F), and 0.5mm (G, H). (J) Protein interactions detected by yeast two-hybrid assays. ++, very strong interaction; +, strong interaction; –, no interaction.

*Actinidia deliciosa* has a high chilling requirement (Wall *et al.*, 2008) and late bud break, and fails to flower under glasshouse conditions. Therefore, to evaluate further the role of *SVP3*, the non-cultivated kiwifruit species *A. eriantha* was chosen for transformation, as it provided advantages including low chilling requirement and early bud break, fast maturity, and prolific flowering in glasshouse conditions (Wang *et al.*, 2006). *Actinidia eriantha SVP3* shares almost identical sequence and expression pattern with the previously studied cultivated kiwifruit species *A. deliciosa* and *A. chinensis* (Supplementary Fig. S1A, B available at *JXB* online; Wu *et al.*, 2012). Six transgenic lines with moderate to high levels of *SVP3* transgene expression and three control lines were generated (Fig. 2A). None of the lines showed any obvious difference during callus formation, growth in tissue culture, and establishment in the soil; similar growth rate and architecture were observed (Supplementary Fig. S1 available at *JXB* online), and in all lines the time of bud break and flowering time (appearance of visible floral buds) were comparable (Table 1). The number of flowers was highly variable and not correlated with transgene expression levels, but the duration of flower development and appearance of flowers were altered significantly in transgenic lines, particularly in the highest expressing Line 1 (Table 1).

**Table 1.** Phenotypic analysis of six lines of 35S:SVP3 transgenic A. eriantha compared with three control linesBud break time represents the number of days from 100% leaf drop to the first visible bud break. Flowering time represents the number of days from the first visible bud break to the first visible flower bud. Anthesis time represents the number of days from visible flower buds to fully open flowers.

*A. eriantha* transgenic lines	Bud break time (d)	Flowering time (d)	No. of flowers	Anthesis time (d)	Petal colour
Line 1	38	25	12	55.1±10.3	White/green
Line 2	32	23	2	49.7±4.1	Light pink
Line 3	38	24	1	43	Light pink
Line 4	38	26	2	47.6±6.8	Light pink
Line 5	32	21	10	46.2±7.6	Light pink
Line 6	35	21	2	45.1±9.3	Light pink
Control 1	35	28	12	27.4±4.1	Pink
Control 2	40	22	35	21.4±5.0	Pink
Control 3	32	24	5	27.8±6.2	Pink

Data are expressed as the mean± SE.

For further detailed analysis, Line 1 and Line 2 were chosen as representatives of high and intermediate *SVP3* transgene expression, respectively. Flower buds in Line 1 and to a lesser extent in Line 2 were not fully enclosed with sepals (Fig. 2B); the number and order of developing floral organs appeared normal (Fig. 2C), but flowers in Line 1 remained smaller (Fig. 2D). Fruit in Line 1 was misshapen and smaller compared with control or Line 2 fruit (Fig. 2E, F) and the transgenic seed was smaller, lighter (Fig. 2G–I), and failed to germinate.

Floral reversion phenotypes observed upon expression of *STMADS* genes in *Arabidopsis* result from interaction with *Arabidopsis* floral homeotic MADS box proteins, particularly AP1 (Fornara *et al.*, 2008; Gregis *et al.*, 2009; Wu *et al.*, 2012). To test if phenotypes observed upon ectopic expression of *SVP3* in *A. eriantha* arose from similar MADS box protein interactions, yeast two-hybrid analysis was performed using previously characterized kiwifruit floral MADS box genes (Varkonyi-Gasic *et al.*, 2011). SVP3 interacted with kiwifruit AG and SEP proteins SEP1, SEP3, and SEP4 with similar strong interaction intensities; a weaker interaction was identified with FUL and FUL-like, but no interaction was detected with AP3, PI, and the negative control CEN (Fig. 2J).

### *SVP3* affects petal colour in transgenic *A. eriantha* by transcriptional repression of key genes in the anthocyanin pathway

**Fig. 3.**
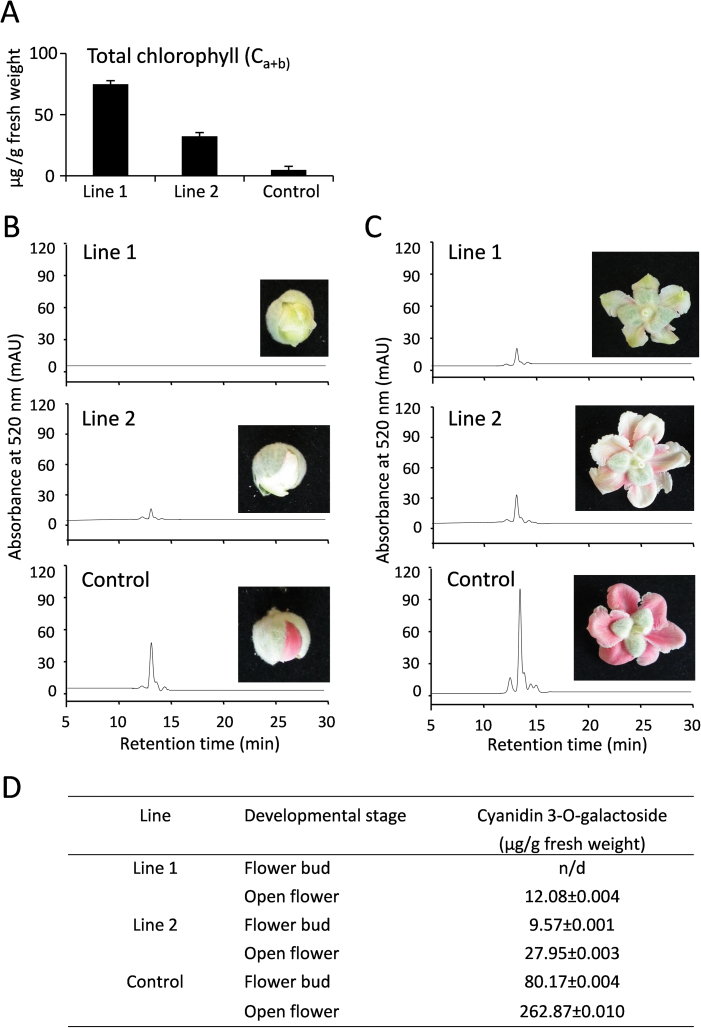
Biochemical analysis of petal pigmentation. (A) Total chlorophyll content in petals of fully opened flowers. Error bars represent the SE of three independent measurements. (B, C) HPLC assay for cyanidin 3-*O*-galactoside correlated with visual phenotypes of flower buds (B) and open flowers (C). (D) Quantification of cyanidin 3-*O*-galactoside using LC-MS. Data represent the means ±SE (*n*=3).

In addition to the described phenotypes, the colour of the petals of *35S:SVP3* lines was dramatically reduced (Fig. 2D, Table 1) and the chlorophyll content was higher (Fig. 3A).

The presence and intensity of the peak corresponding to the major kiwifruit anthocyanin, cyanidin 3-*O*-galactoside, correlated with the visual petal phenotype in flower buds and open flowers (Fig. 3B, C), and further quantification confirmed differential accumulation of cyanidin 3-*O*-galactoside (Fig. 3D) and several minor flavonoid compounds (Supplementary Fig. S2 available at *JXB* online).

**Fig. 4.**
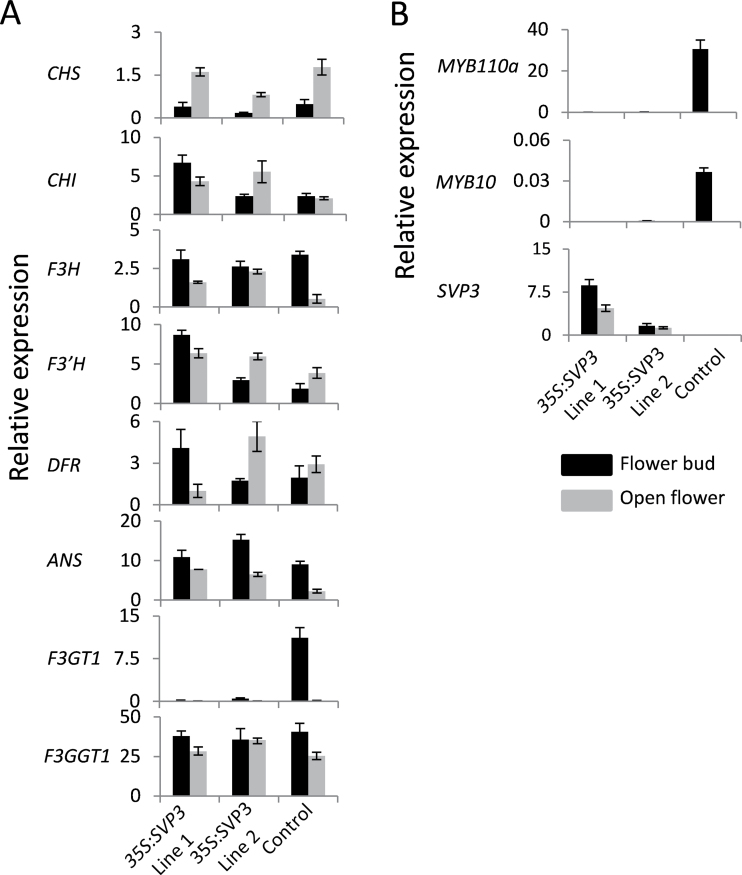
Relative expression of genes responsible for anthocyanin biosynthesis in kiwifruit. (A) Relative expression of kiwifruit flavonoid biosynthetic pathways genes. *CHS*, chalcone synthase; *CHI*, chalcone isomerase; *F3H*, flavonoid 3-hydroxylase; *F3’H*, flavonoid 3’-hydroxylase; *DFR*, dihydroflavonol 4-reductase; *ANS*, anthocyanidin synthase; *F3GT1* and *F3GGT1*; flavonoid 3-*O* glycosyltransferases. (B) Relative expression of regulatory *MYB* genes. Relative expression of *SVP3* is presented below. The expression was normalized against kiwifruit *actin*. Error bars represent the SE of three replicate reactions.

Analysis of the relative expression of the genes encoding enzymes of the anthocyanin biosynthetic pathway (Montefiori *et al.*, 2011) revealed similar expression profiles in representative *35S:SVP3* and control lines, with some variation between flower bud and open flower samples. The exception was the transcript of *F3GT1*, which was absent or barely detectable in flower buds of *35S:SVP3* lines, in contrast to relatively high accumulation observed in the control flower bud (Fig. 4A). Analysis of regulatory genes of the anthocyanin biosynthetic pathway (Fraser *et al.*, 2013) revealed high relative expression of *MYB110a* and low relative expression of *MYB10* in flower buds of the control line. In *35S:SVP3* lines these transcripts were absent or barely detectable, and in the control line both transcripts showed a decrease in open flowers (Fig. 4B).

### *SVP3* does not alter flowering time but affects reproductive development and petal colour in transgenic tobacco

**Fig. 5.**
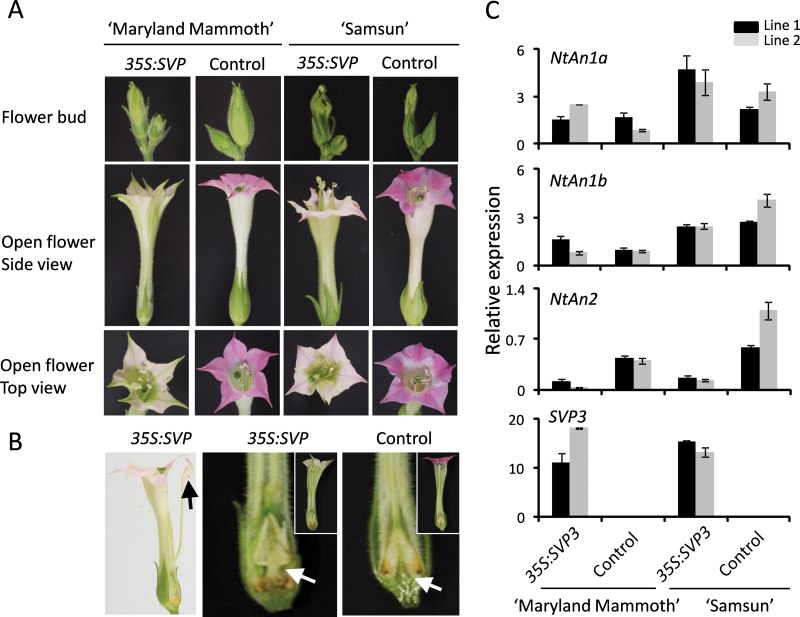
Expression of kiwifruit *SVP3* in transgenic tobacco. (A) Phenotypic analysis of flower buds and open flowers. (B) Floral organ abnormalities: petaloid stamen (black arrow) and ovary position (white arrows). The cross-sections of whole flowers demonstrating colour change are presented in insets. (C) Relative expression of *NtAn1a*, *NtAn1b*, *NtAN2*, and *SVP3* transgene in petals normalized against *Ntα-Tub1.* Error bars represent the SE for three replicate reactions.

To investigate further whether kiwifruit *SVP3* affects reproductive development and anthocyanin accumulation in a conserved manner, transgenic tobacco lines were generated using the *35S:SVP3* construct. A day-neutral tobacco ‘Samsun’ and a short-day variety ‘Maryland Mammoth’ were used. This approach provided the opportunity to establish if *SVP3* affects flowering time in an annual representative of asterids, the clade of eudicots to which the perennial kiwifruit belongs. Eight independent transgenic lines of tobacco ‘Samsun’ and three of ‘Maryland Mammoth’ were generated. Two homozygous T_2_ lines of each cultivar with high transgene expression and two corresponding wild-type control lines were chosen for detailed analysis. No significant differences were observed in vegetative growth and flowering time, but the duration of flower development was extended, flower fertility was diminished, and seed germination was delayed (Table 2). Spike-shaped sepals in transgenic plants failed to enclose flower buds fully, the corolla remained green, and the distal part of the petal was altered in both colour and shape (Fig. 5A). Additional phenotypes were identified, including occasional homeotic conversion of stamen to petal and change in the ovary shape and its position relative to attachment of other floral parts (Fig. 5B). Changes in total chlorophyll, anthocyanin, and flavonoid content and presence of stomata in green petal segments were also noted (Supplementary Fig. S3 available at *JXB* online). To establish if reduced pigment accumulation resulted from transcriptional repression of key regulators of anthocyanin biosynthesis, quantitative PCR (qPCR) analysis of basic helix–loop–helix (bHLH) genes, *NtAn1a* and *NtAn1b*, and the R2R3 MYB gene, *NtAn2* (Pattanaik *et al.*, 2010; Bai *et al.*, 2011), was performed. *NtAn2* was significantly and consistently down-regulated in transgenic lines, while *NtAn1a* and *NtAn1b* expression showed only minor variations (Fig. 5C).

**Table 2.** *Phenotypic analysis of transgenic* 35S:SVP *tobacco ‘Maryland Mammoth*’ *and* ‘*Samsun*’Flowering time was measured as the number of leaves when visible flower buds were observed. Anthesis time represents days from flower buds to fully open flowers. Total number of flowers includes sterile flowers. Seed germination was recorded as number of days to visible germination on half-strength MS medium.

Cultivar	Genotype	Flowering time (d)	Anthesis time (d)	Total flowers	Sterile flowers	Seed germination
Maryland Mammoth	*35:SVP* Line 1	19.5±0.19	7±1.4	27.5±0.5	20.5±2.2	16±0.3
	*35:SVP* Line 2	20.8±1.0	8±0.7	40.7±4.4	28.3±9.2	18±0.2
	Wild-type Line 1	20.9±0.3	5±1.5	27.4±1.6	3.7±0.7	8±0.0
	Wild-type Line 2	22.0±0.6	4±1.2	31.7±3.4	2.5±0.6	8±0.0
Samsun	*35:SVP* Line 1	33.0±1.1	6±1.0	23.7±1.5	13.2±7.2	9±0.2
	*35:SVP* Line 2	42.0±0.8	7±1.4	26.7±3.0	12.1±6.3	11±0.4
	Wild-type Line 1	34.0±0.1	4±0.6	21.7±3.7	2.8±0.4	6±0.0
	Wild-type Line 2	36.0±1.5	4±1.6	24.7±3.8	3.0±0.2	6±0.0

Data represent the mean ±SE of six individuals for each genotype.

## Discussion

### Overexpression of *SVP3* affects reproductive development but not floral transition in transgenic kiwifruit and tobacco

Ectopic expression of the *STMADS* gene subfamily often results in altered flowering time, severe floral abnormalities, delayed flower anthesis, and senescence (Hartmann *et al.*, 2000; Yu *et al.*, 2002; Lee *et al.*, 2007; Fornara *et al.*, 2008; He *et al.*, 2010; Li *et al.*, 2010), and affects duration of endodormancy in woody perennial species (Sasaki *et al.*, 2011). Overexpression of *SVP3* in *A. deliciosa* showed no major effect on vegetative growth and the timing of first visible bud break in glasshouse conditions, although insufficient winter chilling and large variation in the position and number of synchronously breaking buds might have affected the rate of visible shoot outgrowth. Overexpression of *SVP3* in *A. eriantha* and tobacco showed no significant effect on plant growth, bud break, and flowering time, supporting the findings in *A. deliciosa*. However, flower, fruit, and seed development were severely affected. The size and shape of sepals were abnormal and they failed to enclose flower buds fully, consistent with the phenotype observed in *Arabidopsis* (Wu *et al.*, 2012) and similar to other reports (Masiero *et al.*, 2004; Liu *et al.*, 2007; Trevaskis *et al.*, 2007; Fornara *et al.*, 2008). The petals contained vegetative features including increased chlorophyll content and stomata, similar to phenotypes observed upon overexpression of potato *StMADS16* and sweet potato *IbMADS3-1* in tobacco (Garcia-Maroto *et al.*, 2000; Shin *et al.*, 2011). Flower development was slower, resulting in significantly delayed anthesis and an increased proportion of sterile flowers, which failed to produce fruit (Fornara *et al.*, 2008; Li *et al.*, 2010). Additional phenotypes included underdeveloped and misshapen fruit, abnormal seed development, reduced seed germination rate, and extended seed germination, suggesting that *SVP3* affected all stages of reproductive development. The severity of the observed phenotypes during reproductive development, combined with normal vegetative growth, wild-type bud break and flowering time, and relatively high levels of expression in vegetative tissues (Wu *et al.*, 2012), indicate that *SVP3* has a role in the regulatory network of vegetative organogenesis and early stages of floral transition as the key repressor of floral organ development. It is hypothesized that *SVP3* acts to retain vegetative features during plant growth and floral transition, and has to be removed to enable subsequent stages of reproductive development. Ectopic expression phenotypes, combined with constitutive vegetative expression, suggest that there is no involvement of *SVP3* in maintenance of winter dormancy and general growth inhibition. However, SVP3 may have a role in these processes as an interacting partner of other, rate-limiting proteins, and a loss-of-function phenotype would be required to understand *SVP3* function fully.

### *SVP3* interferes with anthocyanin biosynthesis in petals via repression of an anthocyanin-related MYB transcription factor

Overexpression of MADS-box genes is often associated with floral abnormalities, including changes in petal pigmentation. Light green sepaloid petals were observed upon overexpression of *Arabidopsis AGAMOUS-LIKE 20* (*AGL20* or *SOC-1*) in *Arabidopsis* (Liu *et al.*, 2007) and of the mustard *AGL20* orthologue *MADSA* in short-day tobacco ‘Maryland Mammoth’ (Borner *et al.*, 2008). Similarly, expression of *STMADS* genes such as potato *StMADS16* and sweet potato *IbMADS3-1* in tobacco resulted in development of chlorophyll-enriched petals (Garcia-Maroto *et al.*, 2000; Shin *et al.*, 2011). However, these reports did not investigate anthocyanin biosynthesis and accumulation in the petals of transgenic plants, and very little is known about potential MADS-box protein interference with the regulatory genes of the anthocyanin pathway. This study demonstrated that *SVP3* reduced anthocyanin accumulation in petals of transgenic *A. eriantha* in a quantitative manner. The underlying mechanism was dramatically reduced expression of the key structural gene *F3GT1* (Montefiori *et al.*, 2011) and transcriptional repression of regulatory MYB transcription factors, in particular *MYB110a*, the key gene required for kiwifruit petal pigmentation (Fraser *et al.*, 2013). A similar mechanism was observed in transgenic tobacco, where *SVP3* interfered with transcription of the regulatory MYB *NtAn2*.

At this stage, it is unclear if *SVP3* acts by direct or indirect repression of the regulatory MYB transcription. Currently, there is little knowledge about upstream regulators of anthocyanin-related R2R3 MYB expression. Some MADS box genes from the *AP1*/*SQUA* class have been implicated in regulation of anthocyanin accumulation. *IbMADS10* expression correlated with red pigmentation in sweet potato, and ectopic expression resulted in anthocyanin accumulation in transgenic sweet potato callus and transgenic *Arabidopsis* (Lalusin *et al*., 2006, 2011). *VmTDR4* expression was linked with colour development and anthocyanin-related gene expression in bilberry, while silencing of this gene reduced anthocyanin levels and altered expression of the regulatory R2R3 MYB transcription factor (Jaakola *et al.*, 2010). STMADS proteins have a capacity to interact with other MADS box proteins, and interaction with AP1 affected flowering time and flower development in transgenic *Arabidopsis* (Fornara *et al.*, 2008; Gregis *et al.*, 2008; Wu *et al.*, 2012). Therefore, a similar mechanism might be responsible for repression of *MYB110a* and *NtAn2* transcription and reduced anthocyanin accumulation in transgenic kiwifruit and tobacco flowers. The yeast two-hybrid assays showed that SVP3 could interact with kiwifruit AP1/SQUA proteins FUL and FUL-like, and this interaction may interfere with activation of R2R3 MYB transcription. Even stronger interactions were observed with AG and SEP MADS box proteins required for normal flower and fruit development (Varkonyi-Gasic *et al.*, 2011). Therefore, interference with establishment of homeotic MADS box protein complexes, as previously shown in *Arabidopsis* (Fornara *et al.*, 2008; Gregis *et al.*, 2008; Wu *et al.*, 2012), is the likely mechanism by which SVP3 interferes with various aspects of normal reproductive development.

Further functional studies using transgenic kiwifruit and tobacco generated in the course of this study will aid in establishing direct targets and protein partners of SVP3, providing understanding of the pathways and plant processes regulated by SVP3. These plants also provide an exciting opportunity for deeper understanding of the regulation of anthocyanin-related MYB expression in flowers.

## Supplementary data

Supplementary data are available at *JXB* online.

Figure S1. *SVP3* in *Actinidia eriantha*.

Figure S2. LC-MS analysis of flavonoid compounds in petals of *35:SVP3* and control *A. eriantha*.

Figure S3. Analysis of transgenic *35S:SVP3* tobacco.

Supplementary Data
