# Supplementary material for: Overexpression of the kiwifruit SVP3 gene affects reproductive development and suppresses anthocyanin biosynthesis in petals, but has no effect on vegetative growth, dormancy, or flowering time
Source: J Exp Bot. 2014 Jun 19;65(17):4985–95. doi: 10.1093/jxb/eru264 (PMC4144777; doi:10.1093/jxb/eru264)
Supplement: Supplementary Data [file supp_65_17_4985__index.html]

Overexpression of the kiwifruit SVP3 gene affects reproductive development and suppresses anthocyanin biosynthesis in petals, but has no effect on vegetative growth, dormancy, or flowering time — Overexpression of the kiwifruit SVP3 gene affects reproductive development and suppresses anthocyanin biosynthesis in petals, but has no effect on vegetative growth, dormancy, or flowering time — Supplementary Data 

# Overexpression of the kiwifruit *SVP3* gene affects reproductive development and suppresses anthocyanin biosynthesis in petals, but has no effect on vegetative growth, dormancy, or flowering time

## Supplementary Data

Data files

**Files in this Data Supplement:**

- Supplementary Data - Supplementary Data
