# Supplementary material for: Overexpression of the kiwifruit SVP3 gene affects reproductive development and suppresses anthocyanin biosynthesis in petals, but has no effect on vegetative growth, dormancy, or flowering time
Source: J Exp Bot. 2014 Jun 19;65(17):4985–95. doi: 10.1093/jxb/eru264 (PMC4144777; doi:10.1093/jxb/eru264)
Supplement: Supplementary Data [file supp_eru264_jexbot124156_file001.pdf]

Over-expression of the kiwifruit *SVP3* gene affects reproductive development and suppresses anthocyanin biosynthesis in petals, but has no effect on vegetative growth, dormancy or flowering time

Rongmei Wu, Tianchi Wang, Tony McGie, Charlotte Voogd, Andrew C Allan, Roger P Hellens, Erika Varkonyi-Gasic

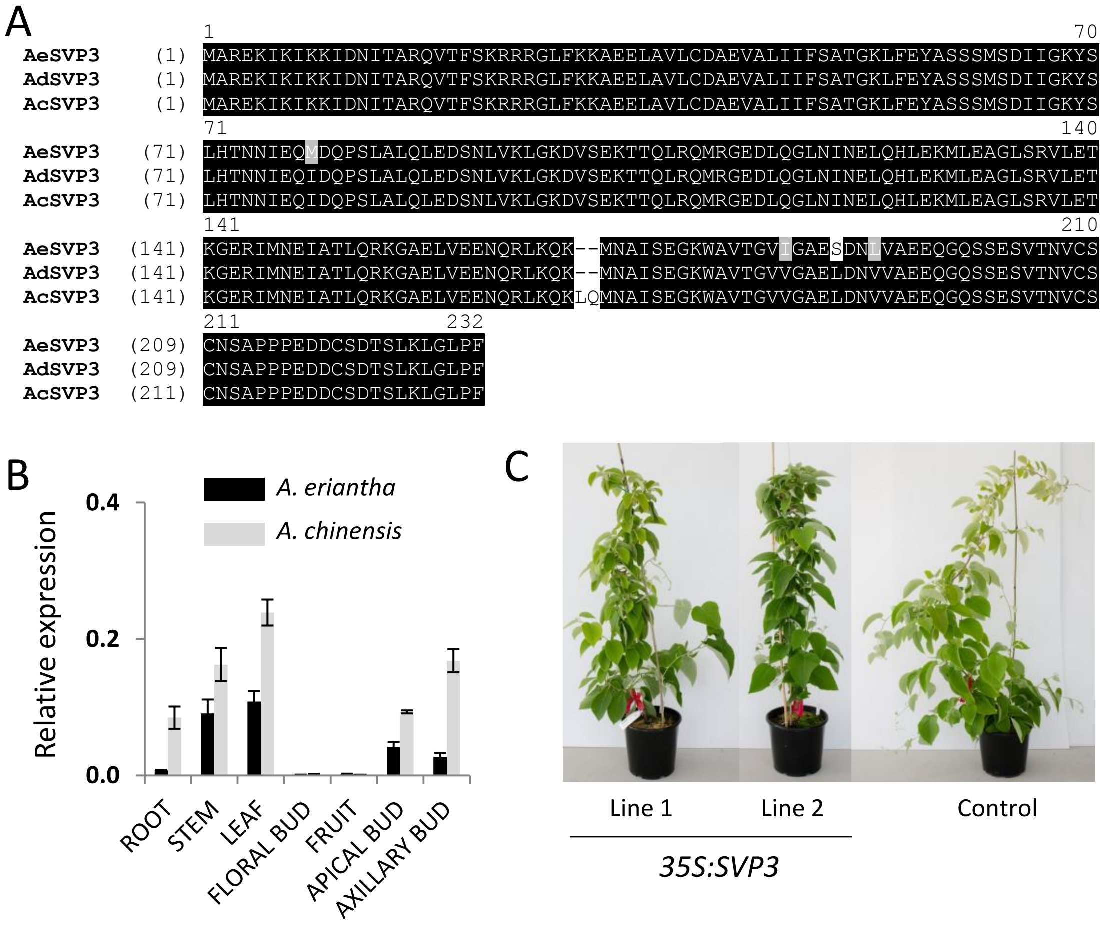

Fig. S1. *SVP3* in *Actinidia eriantha*. (A) Alignment of predicted kiwifruit SVP3 protein sequences. Ae, *A. eriantha*; Ad, *A. deliciosa*; Ac, *A. chinensis*. The amino acid residues in the alignment are coloured according to the following scheme: white on black, identical or conservative residues; white on grey, similar residues, black on white, non-similar or weakly similar residues. (B) Tissue-specific relative expression of kiwifruit *SVP3.* The level of expression was normalised to *Actin*. Error bars represent SE for three replicate reactions. (C) Transgenic *A. eriantha* plants and control plants.

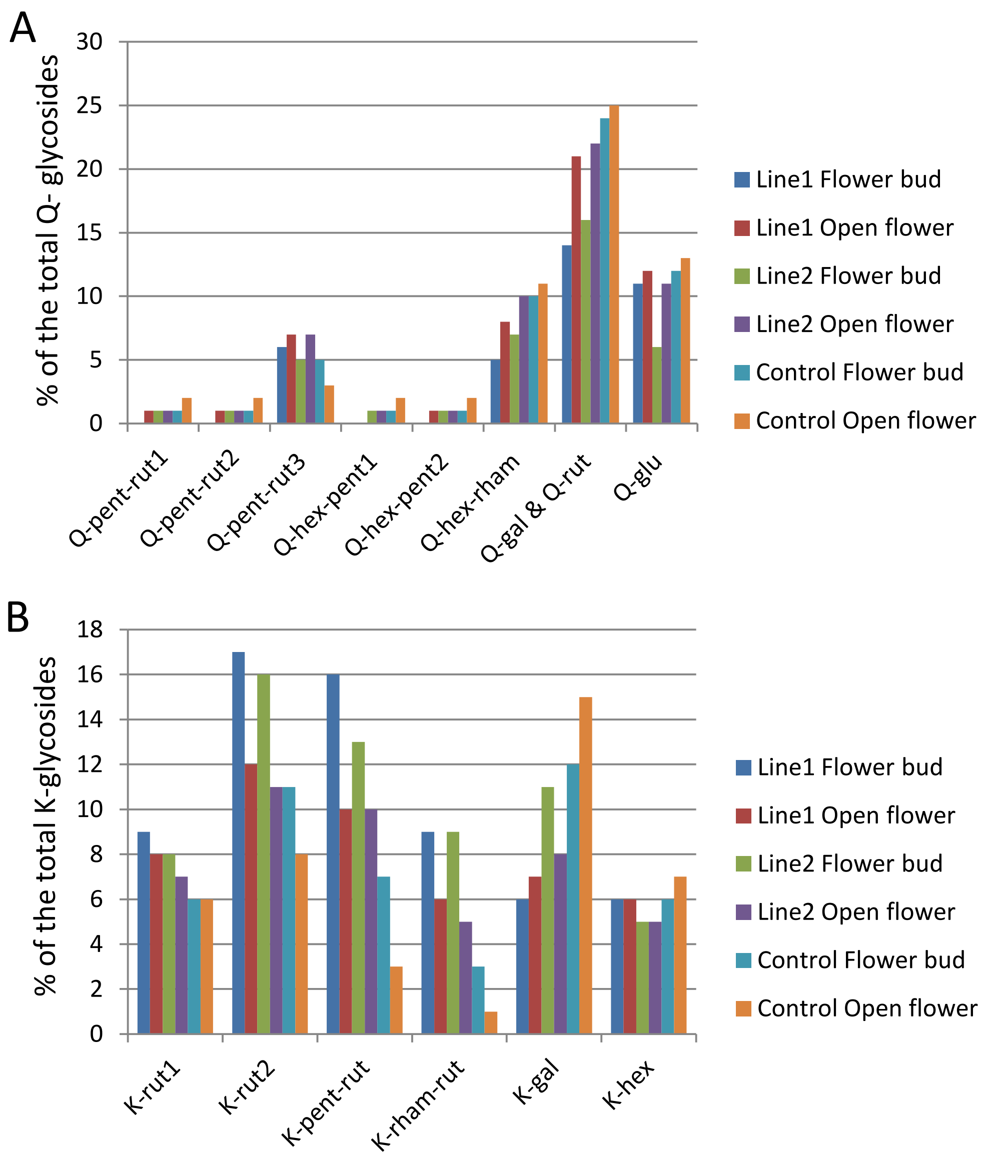

Fig. S2. LC-MS analysis of flavonoid compounds in petals of *35:SVP3* and control *A. eriantha*. (A). Quercetin (Q) glycosides. (B) Kaempferol (K) glycosides. Data represent the peak area and are calculated as a percentage of the total Q- or K- glycosides. rut, rutinoside; pent, pentoside; hex, hexoside; rham, rhamnoside; gal, galactoside; glu, glucoside.

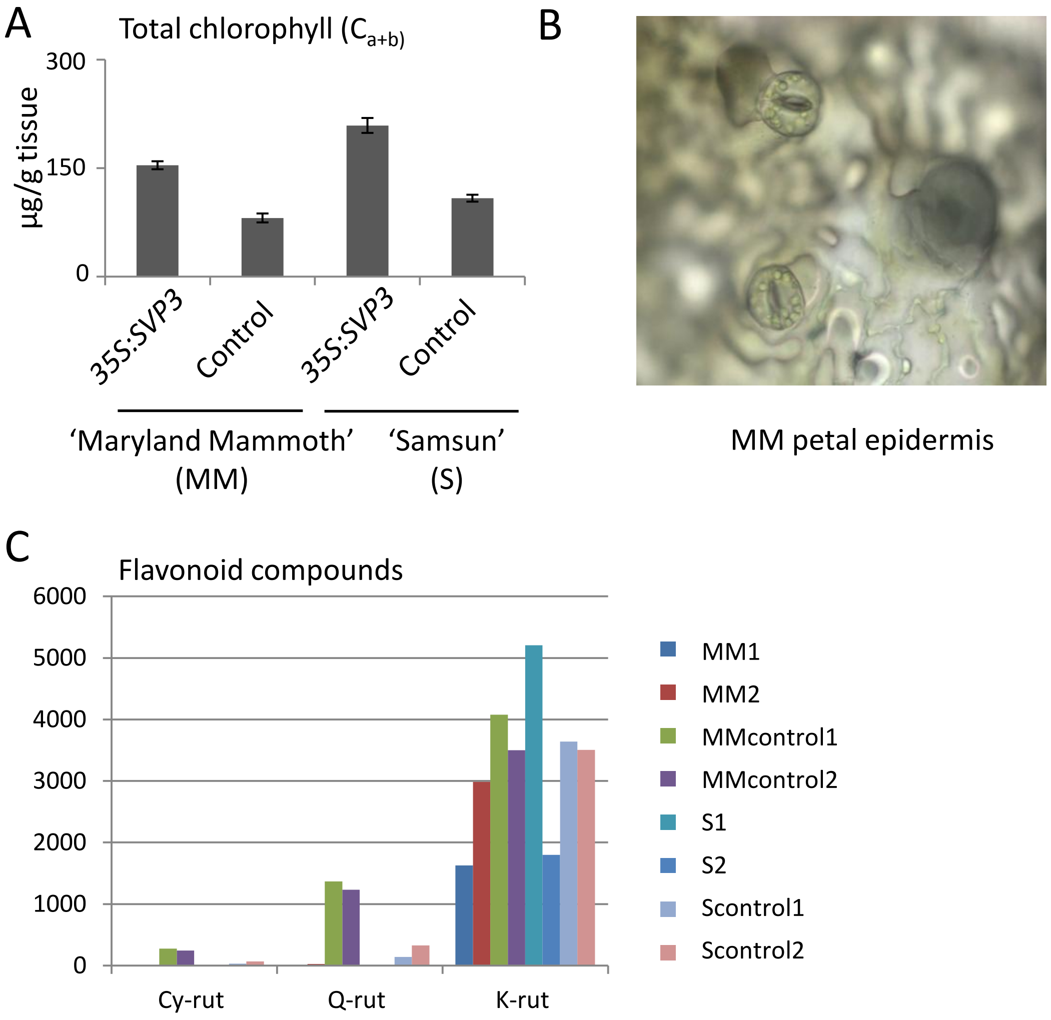

Fig. S3. Analysis of transgenic *35S:SVP3* tobacco. (A) Total chlorophyll content in petals. Error bars represent the SE of three independent measurements. (B) Stomata in the epidermis of *35S:SVP3* transgenic tobacco 'Maryland Mammoth' petals (C) LC-MS analysis of flavonoid compounds in tobacco flowers. Cy-rut, Cyanidin 3-*O*–rutinoside, Q-rut, Quercetin rutinoside, K-rut, Kaempferol rutinoside.
